# On the long-term storage of tissue for fluorescence and electron microscopy: lessons learned from rat liver samples

**DOI:** 10.1007/s00418-024-02334-5

**Published:** 2024-11-27

**Authors:** Gerald J. Shami, Zenan Chen, Delfine Cheng, Eddie Wisse, Filip Braet

**Affiliations:** 1 University of Sydney, School of Medical Sciences (Molecular and Cellular Biomedicine), Sydney, Australia; 2 Australian Centre for Microscopy and Microanalysis, The University of Sydney, Sydney, Camperdown, NSW 2006 Australia; 3 The Victor Chang Cardiac Research Institute, Darlinghurst, NSW 2010 Australia; 4 Department of Internal Medicine Division of Gastroenterology and Hepatology, Maastricht University Medical Centre, Maastricht, The Netherlands

**Keywords:** Aldehydes, Multimodal microscopy, Fixation, Fluorescent labelling, Tissue preservation

## Abstract

Occasionally, tissue samples cannot be processed completely and are stored under varying conditions for extended periods. This is particularly beneficial in interinstitutional studies where a given research setting may lack the expertise or infrastructure for sample processing, imaging and data analysis. Currently, there is limited literature available on the controlled storage of biological tissues in primary fixatives for fluorescence and electron microscopy. In this contribution, we mimicked various tissue storage scenarios by taking different fixation conditions, storage temperatures and storage durations into account. Rat liver tissue was used for its well-known diversity of cellular ultrastructure and microscopy analysis. Fluorescent labelling of actin, DNA and lipids were employed in conjunction with high-resolution electron microscopy imaging. Herein, we tested three different fixative solutions (1.5% glutaraldehyde, 0.4% glutaraldehyde and 4% formaldehyde and 4% formaldehyde) and stored samples for 1–28 days at room temperature and refrigerator temperature. We found that liver tissue can be stored for up to 2 weeks in a 0.4% glutaraldehyde + 4% formaldehyde fixative solution, while still enabling reliable fluorescent labelling and ultrastructural studies. Ultrastructural integrity was eminent up to 1 month using either glutaraldehyde or formaldehyde fixation protocols. When liver tissue is fixed with a mixture of 0.4% glutaraldehyde and 4% formaldehyde and stored at 4 °C, it retains its capacity for electron microscopy analysis for several years, but loses its capacity for reliable fluorescent labelling studies. In conclusion, we demonstrated that liver tissue can be stored for extended periods enabling profound structure–function analysis across length scales.

**Supplementary Information:**

The online version contains supplementary material available at 10.1007/s00418-024-02334-5.

## Introduction

Literature reveals that there is a need for a standardised approach to store fixed tissues for a longer time. Over the years, various reports have presented data of long-term tissue storage before being processed for ultrastructural observations. These data highlighted the need for a better handling of chemically or physically fixed samples stored for extended periods. Selected examples of significant studies include bowel mucosa (Mount 1997), brain matter (Fix 2000), muscle, kidney and hepatic tissue (Dykstra 2002), ocular lenses (Mohamed 2013) and pancreas (Fortunato 2016). These papers reveal that the technical challenges of prolonged tissue storage are not limited to fundamental research, but the same conundrum poses within diagnostic sciences. Evidence indicates that when samples are handled correctly and processed within well-defined timeframes, high quality data can be obtained without major alterations in normal tissue structure owing to improper processing.

The maximal duration for storing chemically fixed tissues under controlled conditions without deterioration of fine morphology remains somewhat unclear. This is particularly relevant in interinstitutional research settings or large (pre-) clinical multidisciplinary studies. In these situations, the type of fixative used, transport time and storage temperature before further processing for electron microscopy (EM) are key factors in preserving structural integrity. Ideally, samples are handled immediately and without interruption for ultrastructural research (Park 2016; Bozzola 2002; Exbrayat 2013). However, not all laboratories have the necessary, complete infrastructure, technical personnel, methods, materials and expertise to process and evaluate the samples properly. Sometimes, even attributed by unexpected experimental findings during the course of the project in which the team realises the need for additional verification at the subcellular level. Therefore, collaboration makes the exchange of samples a necessary prerequisite.

In this paper, we assessed the effect of various routinely used chemical fixative solutions, storage times and temperatures on the fine structural preservation of rat liver tissue. More specifically, we rigorously evaluated the histology and cellular ultrastructure as markers for tissue preservation at the fluorescence and electron microscopic levels. Liver tissue was chosen in this study owing to our extensive experience with its sample processing and data interpretation, as demonstrated in our previous combined (Wisse 2010; Vreuls 2016; Vreuls 2014) and correlative microscopy studies (Shami 2016, 2021, 2024), which detail standard good practices for preparing liver tissue for ultrastructural assessment. Herein, we found that a mixture of glutaraldehyde and formaldehyde solution combined with cold storage provides the best conditions for extended tissue storage before further microscopy investigation. Finally, driven by innate scientific curiosity, we revisited the same samples that had been stored for over 5 years and observed remarkable preservation at the EM level; however, the fluorescent labelling efficiency was lost.

## Materials and methods

### Animals

Wistar rats (10–12 weeks of age) were housed in plastic cages at 21 °C with a 12-h light–dark cycle and were fed and watered ad libitum. All procedures were conducted in accordance with the guidelines and approval of the Animal Care and Ethics Committee of The University of Sydney. Liver samples measuring approximately 2 cm × 2 cm × 0.5 cm were obtained using a sharp razor blade and placed in a petri-dish containing physiological saline solution at 37 °C. Fixation, as described below, should follow as soon as possible (i.e. within seconds).

### Primary fixation and tissue storage

Primary fixation of liver tissue was performed by means of injection fixation as previously described (Wisse 2010). Briefly, the procedure involved gently holding the piece of tissue at a corner using forceps, and slowly injecting the primary fixative (37 °C) using a 25G needle, until discolouration and hardening of the tissue occurred within minutes, as a clear sign of fixation. Three different commonly used fixative solutions were selected for combined fluorescence and electron microscopy investigations: (i) 1.5% glutaraldehyde (Cat. no. C001, ProSciTech Pty. Ltd., QLD, Australia), (ii) 4% formaldehyde (Cat. no. C004, ProSciTech Pty. Ltd., QLD, Australia) and 0.4% glutaraldehyde and (iii) 4% formaldehyde. All primary fixatives were made up in 0.067 M sodium cacodylate buffer (Cat. no. C020, ProSciTech Pty. Ltd., QLD, Australia) containing 1% sucrose (Cat. no. 179949, Sigma-Aldrich, NSW, Australia) and 2 mM calcium chloride (Cat. no. 31216, Riedel-De Haënag, Seelze-Hannover) at pH 7.4. Proceeding injection fixation, tissues were cut into 3 mm × 3 mm × 3 mm blocks and allowed to react in the primary fixative for no more than 20 min (commencing at the time of injection).

Tissue samples were stored under the conditions as summarised in Table 1, and were assessed for fluorescence labelling retention and ultrastructural fidelity. For the experimental storage conditions, tissues were kept at 4 °C and room temperature (RT) for 7, 14, 21 and 28 days. Tissue samples that were not immediately processed (i.e. > 1 day) were kept in 0.1 M sodium cacodylate buffer and 1% sucrose pH 7.4 (washing buffer) if the primary fixative contained glutaraldehyde – given the irreversibility of fixation when used at physiological pH (Okuda 1991; Migneault 2004). Tissues that were fixed in 4% formaldehyde, were stored in the primary fixative owing to the reversibility of formaldehyde cross-linking (Sutherland 2008). The 1-day tissue samples correspond to the control tissue condition as they followed the standard protocols for optimised liver tissue preservation (Wisse 2010; Vreuls 2014). In this instance, tissues were processed exclusively at 4 °C after the injection fixation.

**Table 1:**
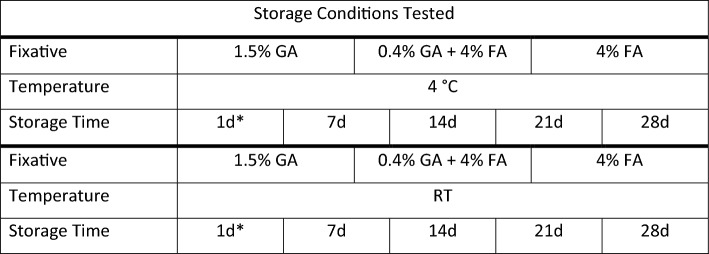
Summary of experimental design illustrating different fixation and storage conditions tested

Summary of the different sample preparation and storage conditions for subsequent specific microscopic assessment. The following fixative solutions were chosen for the following reasons: (i) 1.5% glutaraldehyde as this fixative is preferred for ultrastructural studies owing to its excellent protein cross-linking capabilities, resulting in irreversible bonds; (ii) 0.4% glutaraldehyde and 4% formaldehyde mixture as this this combination is widely accepted for immuno- or fluorescent labelling at both fluorescence and electron microscopy level. It also offers advantages, such as rapid penetration and irreversible cross-linking; and (iii) 4% formaldehyde solution which is commonly used for light or fluorescence microscopy purposes, despite its fast tissue penetration it tends to result in poor preservation of ultrastructure.

*d* days, *GA* glutaraldehyde, *FA* formaldehyde, *RT* room temperature (i.e. stored between temperatures of 20 and 21 °C)

*Corresponds to the control condition and all subsequent tissue sample preparation steps after injection fixation occurred at 4 °C

### Fluorescence microscopy

For fluorescent labelling, tissue blocks were sectioned by means of a Vibratome (Leica VT1200 S Vibratome, Heerbrugg, Switzerland) using the following specifications: knife amplitude 2 mm/second, knife travel speed 1 mm/second, knife angle 9°, section thickness 100 µm. Sections were subsequently placed in a 96-well plate (Cat. no. CLS3599, Corning Costar, Sigma-Aldrich, NSW, Australia) and stained for filamentous actin with 5 units/mL Alexa Fluor^®^ 488 Phalloidin (Cat. no. A12379, Invitrogen Life Technologies, NSW, Australia) for 1 h at RT in darkness. Sections were rinsed with 0.1 M Sorensen’s phosphate buffer (SPB) pH 7.4 (3 × 5 min) and incubated in a filtered saturated solution of Nile Blue A sulphate to label fat (Cat. no. C1291, ProSciTech Pty. Ltd., QLD, Australia) for 1 h at RT in darkness. Tissues were next rinsed in 0.1 M SPB (6 × 5 min) and stained for DNA with 1 µg/mL DAPI (Cat. no. D1306, Invitrogen Life Technologies, NSW, Australia) for 20 min at RT in darkness. Proceeding fluorescence staining, samples were rinsed with 0.1 M SPB (3 × 5 min), placed in an eight-well 1 µm-thick cover glass bottom slide (Cat. no. 80827, Ibidi^®^, Martinsried, Germany) and filled with 0.1 M SPB in preparation of confocal fluorescence microscopy imaging.

Sequential fluorescence images of sections measuring 3 mm × 3 mm × 100 µm were recorded (1024 × 1024 pixels; XY pixel size 201.6 nm; 16-bits per pixel; frame averaging, 8) using a Leica TCS SP5 spectral confocal and multiphoton system (Leica, Heerbrugg, Switzerland) equipped with a Plan 25 × 0.95 N.A. water-immersion objective. For comparative reasons, imaging settings and conditions were held constant for all experimental conditions. Excitation wavelengths for filamentous actin, Nile Blue and DAPI, were 488 nm, 633 nm and 790 nm (multiphoton), respectively. Following fluorescence imaging, samples were transferred to 2 mL Eppendorf tubes and processed for electron microscopy as detailed next.

### Electron microscopy

Proceeding fluorescence imaging, samples were rinsed in washing buffer (3 × 5 min) and post-fixed in 1% aqueous osmium tetroxide (Cat. no. C010, ProSciTech, Pty. Ltd., QLD, Australia) and 1.5% potassium ferrocyanide (Cat. no. 74037, Univar, Australia) in washing buffer for 1 h at RT in darkness. Post-osmication, samples were rinsed with washing buffer (3 × 5 min) and incubated in filtered 1% low molecular weight tannic acid (Cat. no. C081, ProSciTech, Pty. Ltd., QLD, Australia) in washing buffer for 1 h at RT. Tissues were rinsed with ultrapure water (3 × 5 min) and incubated with 2% aqueous uranyl acetate (Cat. no. C079, ProSciTech, Pty. Ltd., QLD, Australia) for 1 h at RT (Shami 2014). Samples were rinsed with ultrapure water (3 × 5 min) and dehydrated in a graded series of ethanol concentrations including 30%, 50%, 70% and 90% for 10 min and 100% in two changes for 10 min, followed by two changes of 100% acetone for 10 min as a transitional solvent at RT. Following dehydration, tissues were progressively infiltrated with one change of medium-grade Epon:Acetone (25%, 50% and 75%) for 3 h. Tissues were placed in 100% Epon (Cat. no. C038, Procure 812, ProSciTech, Pty. Ltd., QLD, Australia) overnight and then into fresh 100% Epon for 3 h. Tissues were placed in the lid of a size 00 BEEM^®^ capsule (Cat. no. 130, Ted Pella, Redding, CA, USA) filled with fresh Epon and polymerised at 60 °C for 48 h.

Silver-gold (~ 70 nm-thick) ultrathin sections were generated using a Leica EM UC7 ultramicrotome (Leica Heerbrugg, Switzerland) equipped with an Ultra 45° DiATOME^®^ diamond knife (Cat. no. 15-US, Electron Microscopy Sciences, Hatfield, PA, USA) and mounted onto 200-mesh copper grids (Cat. no. G200-Cu, Electron Microscopy Sciences, Hatfield, PA, USA). Sections were post-stained with 2% uranyl acetate in 50% ethanol, followed by Reynold’s lead citrate staining for 10 min each.

Inverted backscattered scanning electron micrographs were acquired using a field emission scanning electron microscope (Zeiss Sigma), operating at 3 kV at a working distance of 9.39 mm. The backscattered scanning electron imaging mode was selected relative to transmission electron microscopy owing to higher sample throughput, higher signal-to-noise, particularly at low magnification and its excellent resolving power (~ 5 nm), which was ample for the appraisal of ultrastructural preservation of various organelles (Shami 2019). The microscope was equipped with a Gatan OnPoint^™^ backscattered electron detector, controlled using DigitalMicrograph^®^ Software (v.3.30.2016.0). 16-bit images were acquired to provide information on overall tissue and cell morphology, as well as detailed subcellular features near the nucleus, focusing on mitochondria, endoplasmic reticulum and fine cell inclusions. The following image information pertains to those two different imaging areas, respectively: indicated magnification 1500 × , 15,000 × ; image resolution 8192 × 8192, 4096 × 4096 pixels; pixel dwell time 3 µs; XY pixel size 7.7 nm, 1.5 nm; and field-of-view (XY), 63.14 × 63.14 µm, 6.31 × 6.31 µm.

### Image data

All experiments were repeated at least three times. Image data underwent double-blind interpretation and scored as outlined under Table 2. Microscopy images and metadata were processed using Fiji (v.2.14.0/1.54f) (Schindelin 2012). Images were converted to 8-bit pixel depth and resampled to an image resolution of 2048 × 2048 pixels by means of average pixel binning. Overall image contrast enhancement was performed using histogram normalisation (i.e. linear adjustments to the entire image file). Figure sets were compiled using Adobe Illustrator v. 26.1.

**Ta﻿ble 2:** Summary of different fixation protocols and storage conditions tested with effects on fluorescence retention and ultrastructural preservation quality

Fixative	1.5% glutaraldehyde				
Storage Time	1d	7d	14d	21d	28d
*Fluorescence Microscopy*					
Actin	+ + +				
4 °C		+ +	+ +	+ +	+ +
RT		+ +	+	+ +	+ +
Lipid	+ + +				
4 °C		+ +	±	−	−
RT		+	±	−	−
Nuclei	+ + +				
4 °C		+ + +	+ + +	±	−
RT		+ + +	+ +	±	−
*Electron Microscopy*					
Hepatocytes	+ + +				
4 °C		+ + +	+ + +	+ + +	+ + +
RT		+ + +	+ + +	+ + +	+ + +
Mitochondria	+ + +				
4 °C		+ + +	+ + +	+ + +	+ + +
RT		+ + +	+ + +	+ + +	+ + +

Fixative	0.4% glutaraldehyde and 4% formaldehyde				
Storage Time	1d	7d	14d	21d	28d
*Fluorescence Microscopy*					
Actin	+ + +				
4 °C		+ + +	+ +	+ + +	+
RT		+ + +	+	+ + +	+
Lipid	+ + +				
4 °C		+ + +	+	+ +	±
RT		+ + +	+ +	+	−
Nuclei	+ + +				
4 °C		+ + +	+ +	±	±
RT		+ + +	+ +	±	–
*Electron Microscopy*					
Hepatocytes	+ + +				
4 °C		+ + +	+ + +	+ +	+ +
RT		+ + +	+ + +	+ +	+ +
Mitochondria	+ + +				
4 °C		+ + +	+ + +	+ +	+ +
RT		+ + +	+ + +	+ +	+ +

Fixative	4% formaldehyde				
Storage Time	1d	7d	14d	21d	28d
*Fluorescence Microscopy*					
Actin	+ + +				
4 °C		+ + +	+ +	+ +	+
RT		+ +	−	−	−
Lipid	+ + +				
4 °C		+ + +	+ + +	+	+ +
RT		−	+	+	−
Nuclei	+ + +				
4 °C		+ + +	+ + +	+ +	±
RT		+ +	+	−	−
*Electron Microscopy*					
Hepatocytes	+ + +				
4 °C		+ + +	+ + +	+ +	+ +
RT		+ + +	+ + +	+ +	+ +
Mitochondria	+ + +				
4 °C		+ + +	+ + +	+ +	+ +
RT		+ + +	+ + +	+ +	+ +

Assessment summary of fluorescence staining and ultrastructural preservation in rat liver tissue under various sample preparation and storage conditions. Scoring: Structural preservation was judged on overall tissue and cell integrity (i.e. shortened as ‘structure’), whereas fine structure intactness was evaluated at the organelle level (e.g. mitochondria, RER, cytoplasmic inclusions, etc.) and shortened as ‘ultrastructure’ in the table. (−) poor; (±) (satisfactory); (+) good; (+ +) very good; (+ + +) excellent. Day 1 (1d) samples represent the control condition in which samples were immediately processed as detailed in the Materials and Methods and summarised under Table 1.

*d* days, *RT* room temperature

## Results

In this study, we tested the effects of various fixatives on hepatic tissue, including formaldehyde (FA), glutaraldehyde (GA) and their combination (GA and FA), under different storage conditions and durations, to assess their impact on fluorescence retention and ultrastructural preservation (Table 1).

### Fluorescence microscopy

**Fig. 1:**
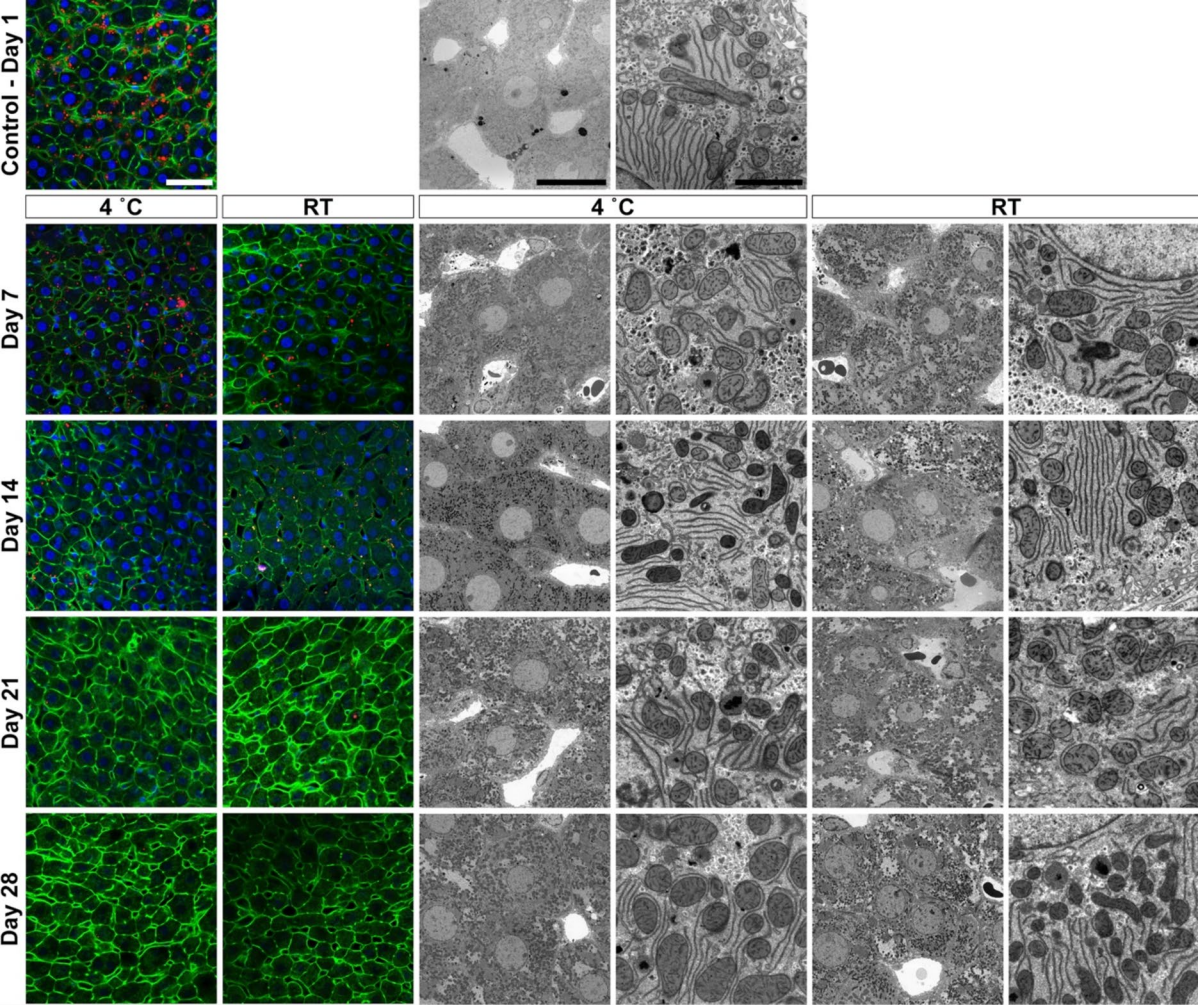
Image panel depicting rat liver tissue fixed in 1.5% glutaraldehyde solution and stored at various temperatures and times. Shown are fluorescence (two coloured image columns on the left) and electron microscopy observations (four grayscale image columns from the middle to the right). Green, filamentous actin; red, lipid; blue, nuclei. Scale bars, fluorescence microscopy 40 µm; electron microscopy 20 µm (left – intermediate magnification) and 2 µm (right – high magnification)

Across the different fixative and storage conditions, the retention of fluorescence varied significantly. Samples fixed with 1.5% GA displayed excellent fluorescence retention for actin, lipid, and nuclei for up to 7 days at both RT and 4 °C. Actin labelling was strong, with only slight decreases observed after 14 days (Table 2, Fig. 1). Whilst lipid staining with Nile Blue A, was initially strong, it began to diminish significantly by day 14, and by day 28, both lipid and nuclear labelling showed noticeable degradation, especially in samples stored at RT. Samples stored at 4 °C exhibited slightly better retention. However, labelling was essentially lost by day 21.

**Fig. 2:**
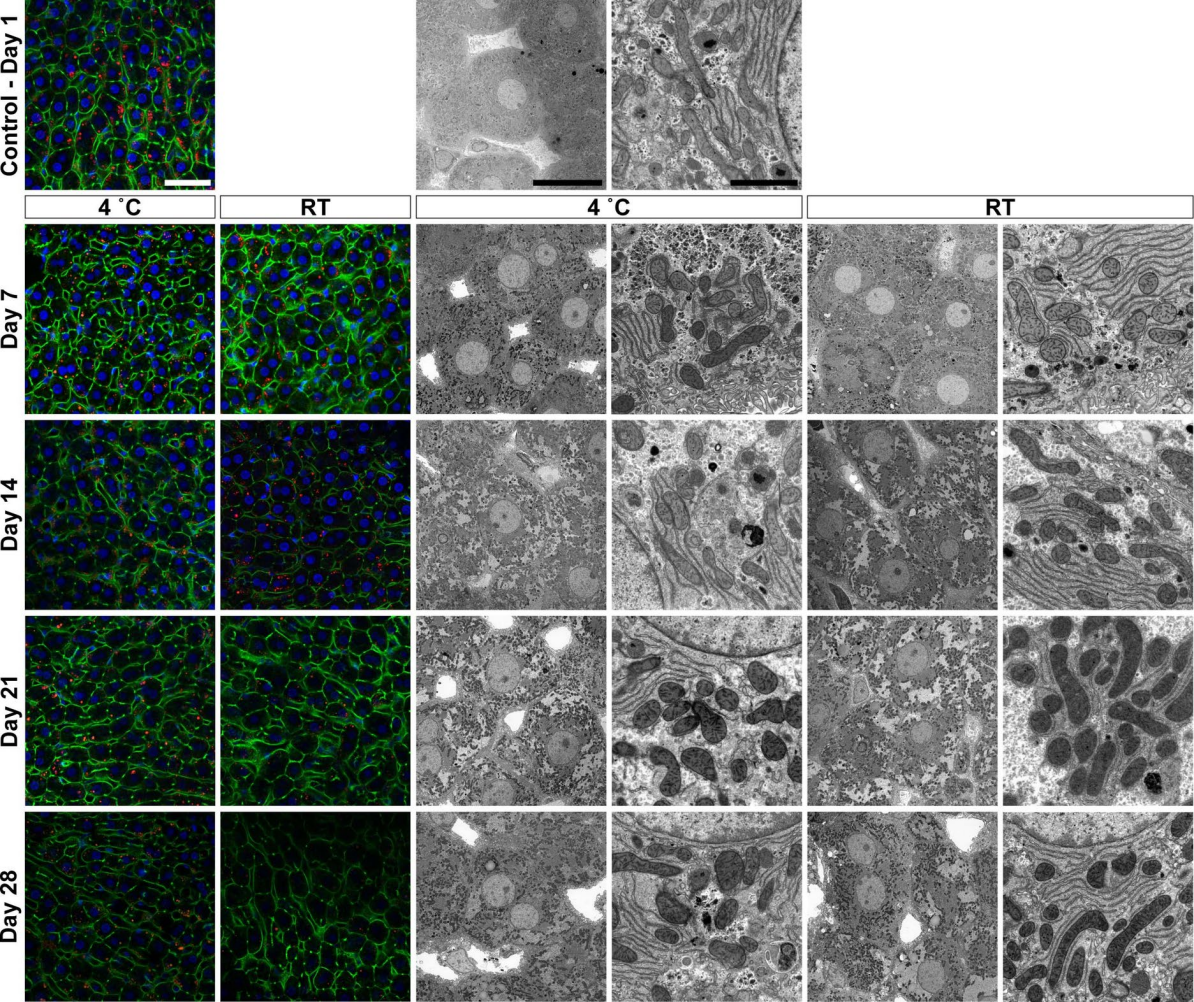
Image panel depicting rat liver tissue fixed in 0.4% glutaraldehyde and 4% formaldehyde mixture, and stored at various temperatures and times. Shown are fluorescence (two coloured image columns on the left) and electron microscopy observations (four grayscale image columns from the middle to the right). Green, filamentous actin; Red, lipid; Blue, nuclei. Scale bars, fluorescence microscopy 40 µm; electron microscopy 20 µm (left—intermediate magnification) and 2 µm (right—high magnification)

In contrast, samples fixed with the 0.4% GA and 4% FA mixture showed more stable fluorescence retention across all conditions assessed. Actin and nuclear staining were particularly well-preserved for up to 14 days, with no significant loss in signal observed at either temperature. Lipid labelling, while more variable, remained detectable up to day 14, with more pronounced fading at RT than at 4 °C (Table 2, Fig. 2). By day 21, fluorescence signals for all markers began to diminish, particularly for lipid, but actin and nuclear labelling were still detectable, albeit weaker. These findings indicate that the 0.4% GA and 4% FA mixture offers superior fluorescence retention over longer storage periods compared with GA or FA alone.

**Fig. 3:**
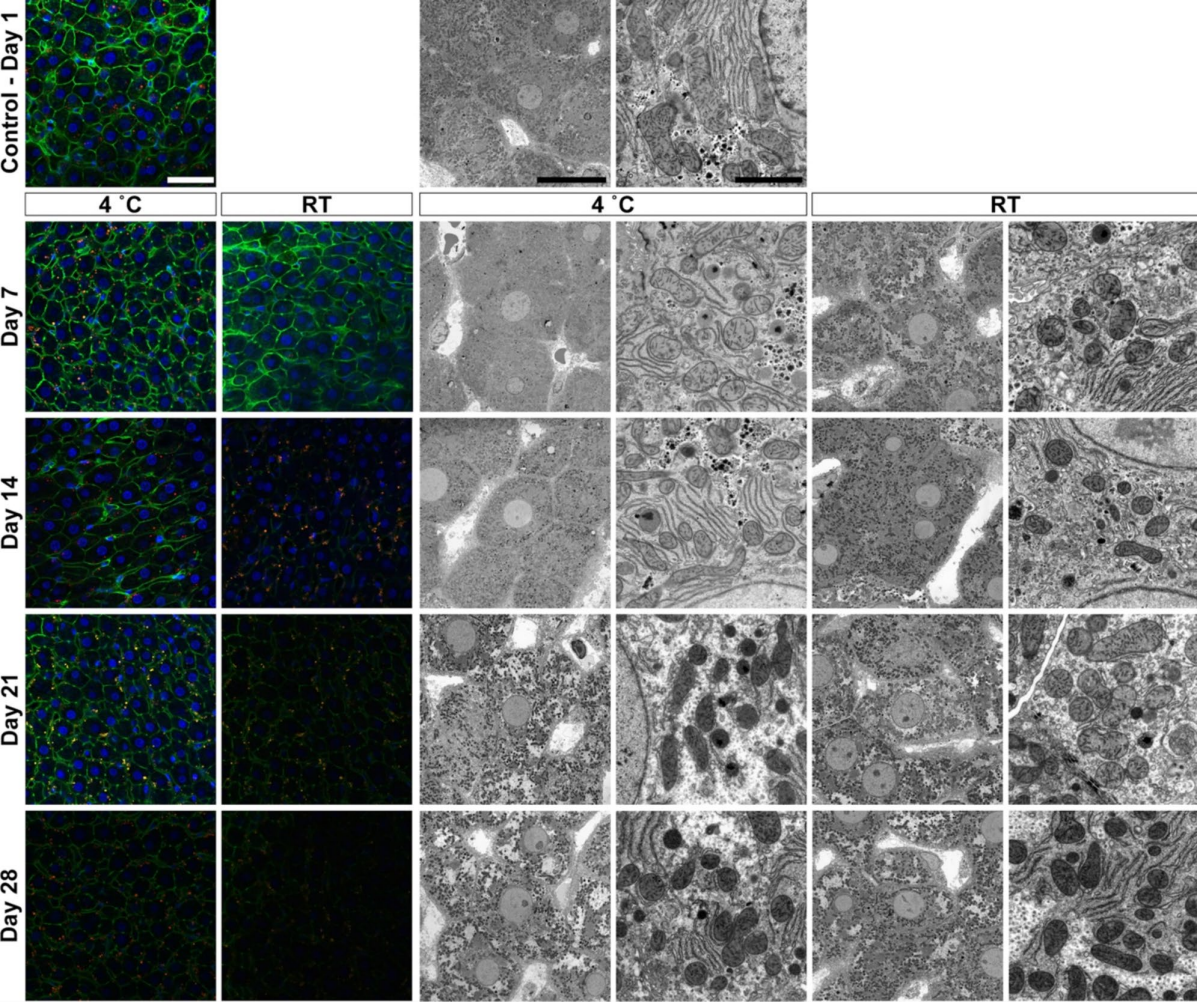
Image panel depicting rat liver tissue fixed in 4% formaldehyde solution and stored at various temperatures and times. Shown are fluorescence (two coloured image columns on the left) and electron microscopic observations (four grayscale image columns from the middle to the right). Green, filamentous actin; red, lipid; blue, nuclei. Scale bars, fluorescence microscopy 40 µm; electron microscopy 20 µm (left – intermediate magnification) and 2 µm (right – high magnification)

In samples fixed with 4% FA alone, fluorescence retention was notably poorer, especially for lipid and nuclear markers. Actin labelling, while initially strong, showed a significant reduction in fluorescence intensity after 7 days, particularly in samples stored at RT, where signal strength was markedly reduced by day 14 (Table 2, Fig. 3). Lipid labelling was the most affected, with signals becoming barely detectable after 7 days at RT. Even at 4 °C, the fluorescence retention for all markers was substantially reduced by day 21, and by day 28, fluorescence signals were minimal to absent.

### Electron microscopy

Electron microscopy analysis revealed that 1.5% GA provided excellent preservation of hepatic ultrastructure, particularly at 4 °C. For samples stored at 4 °C, cellular structures were exceptionally well-preserved even at 28 days, with only minor signs of cytoplasmic degradation (Table 2, Fig. 1). At RT, the ultrastructure remained largely intact up to 21 days, but slight membrane blebbing and mitochondrial swelling were evident by day 28, indicating loss of fine structure.

The 0.4% GA and 4% FA mixture also offered superior preservation of cellular ultrastructure. Cellular morphology was consistently well-preserved across all storage durations, with no significant ultrastructural alterations observed even after 28 days of storage (Table 2, Fig. 2). Mitochondria displayed intact cristae and membrane structures, particularly in samples stored at 4 °C. However, samples stored at RT began to exhibit minor degradation in mitochondrial morphology by day 21, although hepatic parenchymal cells remained largely intact.

In contrast, the use of 4% FA alone resulted in poorer ultrastructural preservation. By day 14, hepatic parenchymal cell membrane integrity began to show signs of degradation, particularly in samples stored at RT (Table 2, Fig. 3). Mitochondrial swelling and loss of cristae were more pronounced, and by day 21, these changes were evident at both RT and 4 °C. While formaldehyde is a widely used fixative for light and fluorescence microscopy, its limitations for long-term ultrastructural preservation were evident in this study.

A notable finding from this study was the surprising ultrastructural preservation observed in samples stored in the 0.4% GA and 4% FA fixative at 4 °C for extended periods. When re-examining samples stored for over 5 years under these conditions, electron microscopy revealed that hepatic parenchymal cells and mitochondria were still well-preserved, though fluorescence labelling was no longer feasible, as expected. The aldehyde-based cross-linking, while stable for ultrastructural preservation, likely underwent reversibility over time, leading to the loss of fluorescent marker binding (Supplement 1). This observation contrasts with samples stored in 1.5% GA or 4% FA, which exhibited a marked decline in both fluorescence and ultrastructural integrity after prolonged storage. The samples fixed in 0.4% GA and 4% FA mixture at 4 °C not only maintained their ultrastructural integrity for years but also showed minimal cytoplasmic degradation, reinforcing the efficacy of this fixative for long-term storage, especially when electron microscopy is the primary investigative method.

## Discussion

Preparing samples for electron microscopy investigation requires adherence to the general principles of tissue processing (Nasser Hajibagheri 1999), including the careful selection of fixatives, appropriate incubation times and recommended temperatures (Park 2016). However, this is not always practically feasible, especially when there is a need to access tissue repositories at a later stage (Grizzle 2010). This situation mainly arises in inter-institutional collaborations or diagnostic settings and in clinical cohort studies.

Tissues are also often physically fixed and stored in liquid nitrogen, kept in chemical fixative solutions at controlled temperatures, or retained as paraffin-embedded samples in biorepositories. The need for well-defined protocols on how to handle prolonged stored tissue samples is especially acknowledged in diagnostic EM or biomarker imaging analysis (e.g., (Mount 1997; Ghadially 1985; Neumeister 2014)).

An important consideration in sample preparation is the successful fixation of tissues, which directly impacts the quality of structural preservation and final characterisation. The choice of fixation method, such as immersion or perfusion, depends on the sample’s nature and the study’s objectives (Watkins 2013; Bozzola 2002). In this investigation, injection-mediated perfusion was selected due to its very rapid action on all different cells in the tissue resulting in more uniform fixation, better morphological detail and reduced artefacts (Vreuls 2014; Wisse 2010). Conversely, immersion fixation is more straightforward and suitable for cell suspensions or easily accessible specimens (Wisse 2010). Similarly, the fixative vehicle (i.e. buffer) plays a critical role in the success of tissue fixation in sample preparation. The choice of vehicle influences the penetration, distribution and overall effectiveness of the fixative (Maunsbach 1966; Rasmussen 1974). Ideally it should be isotonic with the sample and have an excellent buffering capacity to maintain structural integrity and minimise the formation of artefacts. The use sodium cacodylate was selected in this study based on its well-established effectiveness in ultrastructural liver research and its expedience in minimising the growth of microorganisms during long-term storage (Stadtländer 2004). Other commonly used buffers, such as phosphate buffered saline, may not be suitable for long-term storage because they are known to be prone to microbial contamination, particularly when stored at room temperature.

Despite recognising the need to revive long-term stored tissue samples for microscopic assessment – especially those stored in fixatives – available literature on this topic is relatively sparse. Most existing studies focus on practical solutions for tissue recovery from cryo-stored tissue (e.g. Fortunato 2016) or paraffin-embedded histology samples (e.g. Graham 2007). However, the exceptional paper by Dykstra and colleagues (Dykstra 2002) stands out as the only dedicated study addressing the challenges for processing tissue samples that have been stored in primary fixative solutions for extended periods. The authors demonstrated that satisfactory to excellent outcomes, with minimal structural degradation can be obtained in muscle (17 years), kidney (7 years), small intestine (3 years) and liver tissue (3 years). They found that a mixture of four parts formaldehyde to one part glutaraldehyde [i.e. McDowell Trump’s fixative (McDowell 1976)], followed by subsequent storage at 4 °C, is optimal for subsequent light and electron microscopic analysis. This finding underpins our own electron microscopy observations, where combined use of formaldehyde and glutaraldehyde yielded the best results (i.e. compare Fig. 2 with Figs. 1 and 3; Table 2). Furthermore, excellent preservation of liver tissue ultrastructure was achieved even after more than 5 years of cold storage under these fixation conditions, though fluorescence signals were limited to background fluorescence (see Supplementary Information). Notably, the supplementary data were serendipitous side observations made when we resumed the present study after the laboratory closure to the coronavirus disease 2019 (COVID-19) pandemic (Korbel 2020).

Furthermore, we gathered insights on the optimal temperatures for storing liver tissue in various primary fixatives (Table 1). Room temperature (RT) and cold storage (4 °C) were chosen on the basis of standardised protocols for tissue preparation for microscopy (Collan 1976; Bozzola 2002; Watkins 2013). We also included fluorescent labelling experiments, which provide a comprehensive view of overall tissue preservation and indicate what one can expect in terms of morphology preservation at the electron microscopy level. These staining experiments evidently also add value by demonstrating the suitability of long-term stored tissue at different temperatures for combinatorial fluorescent labelling studies (see the left two columns of Figs. 1, 2, 3). We used common biomolecular probes for DNA, actin and lipid that enable the assessment of subcellular constituents and structural preservation (Timmers 2002). In general, we found that temperature was not the primary factor determining overall fluorescent labelling efficiency and ultrastructure retention within the first week of storage for all the fixative solutions tested. However, formaldehyde alone scored the lowest of the three fixative solutions tested, though still yielded relatively acceptable results (Table 2). This provides ample time for sample transport between locations via mail courier services for example. For storage longer than 1 week, the best outcomes at the fluorescence and electron microscopy level were obtained by using the glutaraldehyde-formaldehyde mixture (Fig. 2), irrespective of whether the samples were stored at RT or cold.

By scoring the outcomes of our different experiments (Table 2), we recommend a cut-off time for labelling experiments between 7 and 14 days. This recommendation is based on the significant variations observed in labelling outcomes, particularly when either glutaraldehyde (Fig. 1) or formaldehyde fixative was used in isolation (Fig. 3). Although one might interpret ‘acceptable’ staining outcomes up to 28 days of storage – especially when using glutaraldehyde-fixed tissues stored at room temperature or 4 °C that were subsequently labelled for filamentous actin (Figs. 1) – we strongly discourage to take any labelling experimentations into consideration in ‘long-term’ aldehyde-stored tissue. Indeed, it is known that histochemistry staining outcomes is highly dependent upon the type and duration of fixative employed (Howat 2014). This, is also underpinned by our previous observations that the use of different fixatives at different temperatures results in different labelling outcomes (Vekemans 2004).

We have no direct explanation for the observed staining variability from day 14 onwards, but hypothesise that reversibility of aldehyde binding to tissue components is a major contributing factor (Sutherland 2008; Helander 1994; Hewlett 2002). Supporting this assumption is the work by Helander, who mimicked routine surgical pathology settings using rabbit liver tissues. This work demonstrated formaldehyde reversibility over a period of 6–26 days, during which nearly 90% of the aldehyde binding disappeared from the tissues (Helander 1994). This variability in staining outcomes owing to aldehyde-binding reversibility can also likely be explained, because when glutaraldehyde was used, it was stored in buffer, unlike those fixed with formaldehyde alone. Furthermore, our labelling outcomes on rat liver tissues that were stored for years seem to support the assumption of ongoing reversibility – and hence loss – of aldehyde groups that firmly cross-link proteins (see Supplementary Information). On the other hand, time-dependent chemical reactions, such as those involving glutaraldehyde and free aldehydes reacting with stains, should not be excluded, even though these reactions are presently unknown to us.

The data presented herein, and literature discussed so far, primarily relate to the application of aldehyde-based fixatives. However, various other methods, such as cryogenic fixation and alternative chemical fixation approaches, have been developed for preserving, storing and reviving liver tissues. While each method achieves adequate outcomes, they often require specialised and costly equipment compared to the methodology outlined in this work (Prentø 1997; Vogels 1993; Venter 2013; Galhuber 2021). Briefly, Prentø demonstrated that frozen rat liver tissue stored at −80 °C remains suitable for up to 14 days for ultrahistochemical and ultrastructural studies when swiftly thawed in glutaraldehyde-formaldehyde based Karnovsky fixative (Prentø 1997). Vogels validated and extended these findings in rat liver tissue containing colon carcinoma metastases, showcasing good preservation of liquid nitrogen-frozen tissue stored at −80 °C for up to a year, which was subsequently fixed with glutaraldehyde for further processing for electron microscopy (Vogels 1993). In a related study, Venter et al. highlighted the efficacy of storing mouse liver tissue for up to three weeks in glutaraldehyde and formaldehyde solution, followed by high-pressure freezing (Venter 2013). However, the authors preferred immediate high-pressure freezing as the optimal method for ultrastructural preservation. Reflecting upon these studies, it is evident that a storage time of 2–3 weeks is acceptable, aligning with our present findings (Table 2 and Fig. 2).

Final considerations worth noting include the importance of minimising the time between harvesting biological tissue samples and their exposure to the primary fixative (Bozzola 2002). Previous studies on liver tissue have shown that this time should ideally be reduced to seconds rather than minutes to prevent autolysis of the samples (Vreuls 2014; Wisse 2010). Therefore, when long-term stored tissue samples are part of the experimental planning, proper initial fixation, as described herein, forms the foundation for a successful outcome with prolonged stored liver tissue. Lastly, owing to the increasing awareness of the hazardous nature of aldehyde-based fixatives (Buesa 2008), ongoing research seeks to identify safer and equally effective chemical compounds, such as RCL2^®^, TAG-1^™^ and Glycoxal (De Martino 2022; Peeler 2024). Ideally, these alternatives should offer stable, non-reversible fixation for long-term storage and preserve protein integrity for staining and immunolabelling. However, until a truly viable substitute is developed, aldehyde-based fixatives, used since the early days of microscopy, are likely to remain in use.

## Conclusions

We put forward that liver tissue prepared by injection fixation can be stored for up to 2 weeks in a 0.4% glutaraldehyde and 4% formaldehyde fixative solution, allowing for fluorescence and ultrastructural studies with a high degree of confidence. For electron microscopy, excellent ultrastructural preservation can be anticipated for up to a month when using either glutaraldehyde or formaldehyde fixation protocols. Furthermore, tissue fixation with a mixture of 0.4% glutaraldehyde and 4% formaldehyde, followed by storage at 4 °C, ensures structural intactness at the electron microscopy level for years. On the basis of our findings, worldwide transport of cell and tissue samples that retain their ultrastructure and staining capabilities is feasible, especially when rapid transport can be ensured.

## Supplementary Information

Below is the link to the electronic supplementary material.

Supplementary file1 Supplement 1. Figure sets show the outcomes of rat liver tissue stored for 6-years, following the same experimental sample preparation protocols as summarised under Table 1. Using the same scoring scale as detailed under Table 2, the 4 °C and 1.5% glutaraldehyde (GA) would surprisingly receive a ‘+/-’-score after 6 years of storage and looks very similar under fluorescence imaging conditions to the 28 days sample preparation and 4 °C and 1.5% GA condition (for comparison, see Fig.1). Electron microscopy examination disclosed a certain degree of variation depending on the fixative solution used. Mixture of glutaraldehyde and formaldehyde (FA) undoubtedly excels (++) while storing under either GA or FA revealed the loss of either cellular content or structure. Note: Green, filamentous actin; Red, lipid; Blue, nuclei. Scale bars, fluorescence microscopy 40 µm; electron microscopy 20 µm (left—intermediate magnification) and 2 µm (right—high magnification). (TIF 10112 KB)

## Data Availability

The datasets generated during and/or analysed in the current study are available from the corresponding author upon reasonable request.
