# Supplementary figures and images for: On the long-term storage of tissue for fluorescence and electron microscopy: lessons learned from rat liver samples
Source: Histochem Cell Biol. 2024 Nov 27;163(1):12. doi: 10.1007/s00418-024-02334-5 (PMC11602835; doi:10.1007/s00418-024-02334-5)

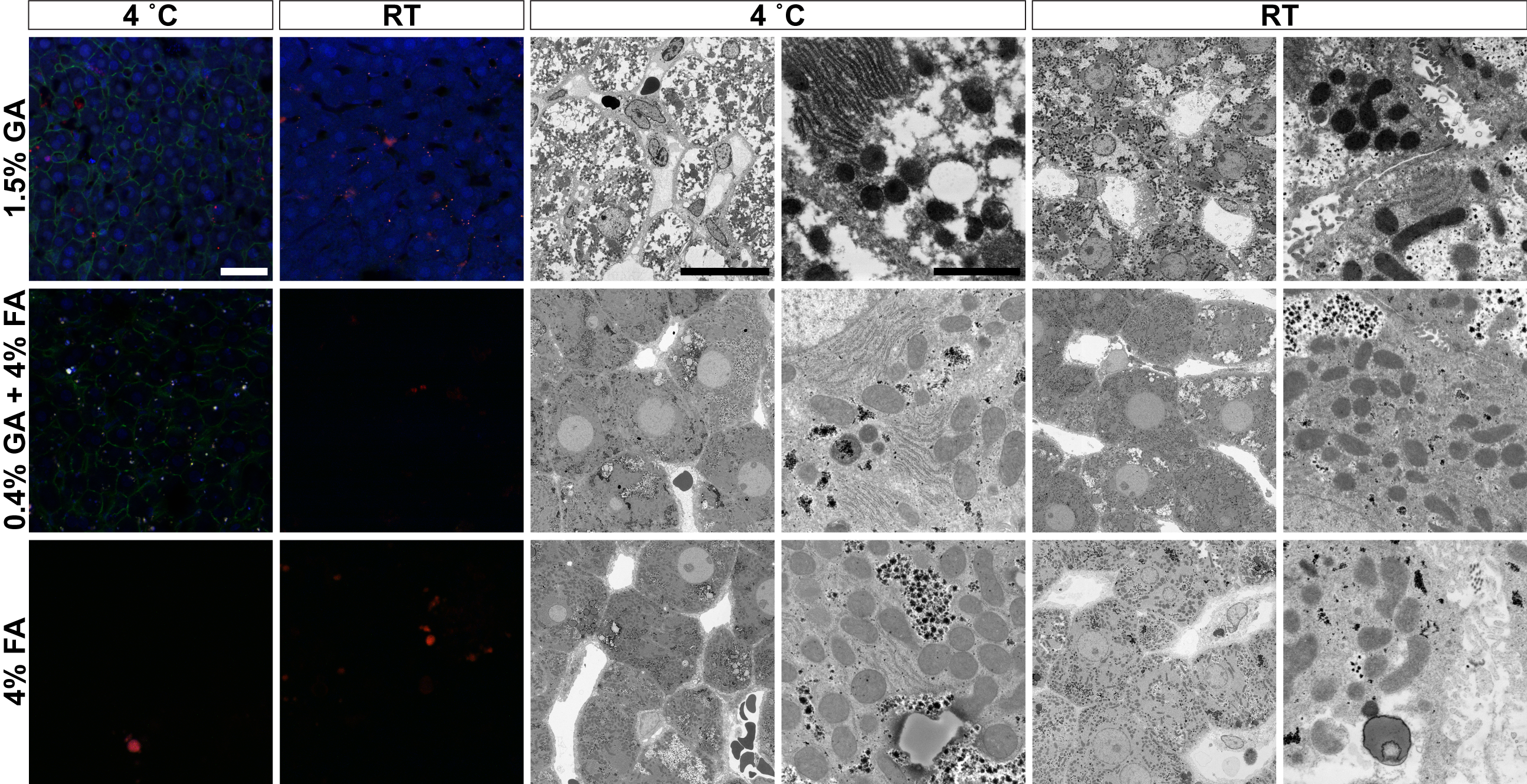

Supplement: Supplementary file 1 — Supplementary file1 Supplement 1. Figure sets show the outcomes of rat liver tissue stored for 6-years, following the same experimental sample preparation protocols as summarised under Table 1. Using the same scoring scale as detailed under Table 2, the 4 °C and 1.5% glutaraldehyde (GA) would surprisingly receive a ‘+/-’-score after 6 years of storage and looks very similar under fluorescence imaging conditions to the 28 days sample preparation and 4 °C and 1.5% GA condition (for comparison, see Fig.1). Electron microscopy examination disclosed a certain degree of variation depending on the fixative solution used. Mixture of glutaraldehyde and formaldehyde (FA) undoubtedly excels (++) while storing under either GA or FA revealed the loss of either cellular content or structure. Note: Green, filamentous actin; Red, lipid; Blue, nuclei. Scale bars, fluorescence microscopy 40 µm; electron microscopy 20 µm (left—intermediate magnification) and 2 µm (right—high magnification). (TIF 10112 KB) [file 418_2024_2334_MOESM1_ESM.tif]
